# Medical Opinion and Movement

**Published:** 1909-03-27

**Authors:** 


### Medical Opinion and Movement.

THE Pancreatic Reaction associated with the name of Dr. P. J. Cammidge proved in its original form very unsatisfactory, and was subjected to severe criticism. The amended test as published by its author in 1907 has been apparently much less fully controlled, possibly owing to the disrepute into which the first process fell. Such independent research as has hitherto been published seemed to bear out only very uncertainly and partially the claims made by Dr. Cammidge. In these circumstances, therefore, the investigations of Dr. Goodman, published in the *Annals of Surgery*, are of much interest. This author investigated 62 cases of abdominal disease, and he obtained a positive reaction ten times. In seven of these ten the patient was proved either by operation or autopsy to be the subject of pancreatic inflammation, and in another death was considered undoubtedly due to acute pancreatitis, though confirmation by autopsy was not obtained. In the other two cases, of diabetes and gastroptosis respectively, a pancreatic lesion is considered to have been highly probable. There were but four negative cases in which a lesion of the pancreas was diagnosed; two of these were cases of malignant disease, in which the test is always negative, the other two of exceedingly mild pancreatitis. The findings of this author agree all through much more closely with those of Dr. Cammidge than with those of some of his critics. The test is, he says, not pathognomonic, nor did its discoverer ever claim as much, but it is " strongly suggestive "; and he adds in a note that extended observations confirm him in this belief.

PROFESSOR NICOLL, of Glasgow, describes in the same issue his method of dealing with Carcinoma of the Penis, on the principle of removing in one mass the disease, the glands towards which extension is likely, and the lymphatic tract connecting the two. Carcinoma in this region is almost always an epithelioma originating on the surface of the glans or prepuce; and the lymphatics of the anterior half of the penis pass almost wholly along the dorsum to the superficial inguinal glands, and secondarily thence to the deep inguinal glands on the brim of the pelvis. Only late in the disease does the growth infiltrate the corposa cavernosa and thus infect the subpubic and intrapelvic glands. Accordingly in all but very advanced cases it is recommended that a clean sweep of the growth and lymphatic tract be made by a strictly Y-shaped incision whose arms extend along the groins nearly to the anterior superior iliac spines. The leg of this incision is carried along the dorsum of the proximal fourth of the penis, and there merges in a loop which encircles obliquely the penis: a bladder staff is inserted at the beginning of the operation, and this last incision is skin deep only. The whole of the fat and glands are then dissected down from each groin, beginning on the under side so as to secure the superficial epigastric and circumflex and the external pudic arteries. If thought necessary the deep glands can also be dealt with by opening the femoral sheath. The dorsal arteries and vein are then tied close to the pubes; the rest of the operation follows on established lines, the only noteworthy point being drainage of the groins, which is necessary for lymphorrhea.

PRE-ECLAMPTIC Toxæmia is exhaustively discussed in the *American Journal of Obstetrics* by Dr. Skeel, who has studied the recent literature very thoroughly, and presents a full bibliography. His main conclusions are, that a trace of albumin in the urine is of no import, for it is present in a large proportion of normal pregnancies, and its absence is no proof that there is no toxæmia. Albumin in considerable quantity, especially if increasing, is a strong indication of mischief; casts and diminished quantity are confirmatory. Blood pressure, to be of any use, must be instrumentally determined. There is normally a slight rise in the last two months of pregnancy, but any pressure over 150 mm. should be regarded as a serious matter. During labour the blood pressure is not to be depended on, but it should afterwards quickly regain the normal. In pre-eclamptic toxæmia it is believed to be always elevated. The leucocyte count is also held to be of value. Excluding the leucocytosis of digestion, inflammation, hæmorrhage, labour, etc., the count shows in pre-eclamptic states a rise of 50 per cent. above the normal. Ophthalmoscopic examination in all such cases is imperative; indeed, one authority says it is called for as a routine measure in all cases of pregnancy. In early pregnancy any demonstrable ocular lesion demands evacuation of the uterus; in the last two months the symptom, though of itself unfavourable, must be compared with the other findings before treatment is decided on.

BUE and Voiron have communicated to the Obstetrical Society of Paris a very complete study of Jaundice in the New-born. The authors recognise two forms of the disease—a symptomatic variety and an idiopathic form of obscure causation. This latter is characterised by its mildness, by its cyclic evolution into three periods—the red or pre-icteric period, the yellow or period of established jaundice, and the period of decline, as well as by the absence of bile in the urine. This so-called idiopathic jaundice would seem to be the result of an extensive destruction of red blood-cells, as can be proved by an examination of the blood of the patient. The treatment of this variety of the condition is simple, consisting in careful surveillance over the feeding of the child, accompanied by the use of tepid baths, and rectal injections of boiled water. Symptomatic jaundice may be classified according to its methods of origin. It may arise from biliary obstruction, in which case it is unpreventable, or it may be the result of infection either through the umbilical cord or from the intestine. The prognosis in such cases is grave. They are best prevented by careful aseptic treatment of the cord and care in the nursing of the child. When once established, the jaundice is best treated by fomentations applied to the cord and a diet largely consisting of water. The stomach and intestines should be washed out frequently and small doses of serum injected subcutaneously by way of nourishment. Another variety of jaundice is the result of syphilis, which is best treated by a vigorous application of the appropriate remedies.

A PECULIAR Affection of Certain Bones in Young Children is described by Dr. A. Köhler. The child complains of more or less intense pain in the middle of the dorsum of the foot, especially in the region of the scaphoid bone. The pain is felt during the day and also at night. In one case there was also pain in the knees about the patella. The children appear to be healthy, and show no trace of rickets. Pressure over the scaphoid causes pain, but otherwise nothing abnormal is revealed on ordinary clinical examination. The *x-rays*, however, shows the scaphoid much diminished in size, so as to be only half or a quarter the normal dimensions, with an irregular serrated contour. There is no clear demarcation between the compact and spongy bone and the mineral content appears to be doubled or quadrupled. In the patient with the knees also affected the patellæ showed the same changes. Under the influence of rest the morbid condition completely cleared up, but only after a duration of one and a half to two years. The examination by the *x*-rays also confirmed the complete disappearance of the affection. The author has encountered three such cases in the course of the last few years, so that he is led to believe that the condition is by no means uncommon. He is unable to offer any explanation as to its pathology, as it in no way suggests rickets, osteomyelits, tuberculosis, or infantile osteomalacia.

THE operation of Direct Blood Transfusion is perhaps more in vogue in America than elsewhere. This is probably due to the attention devoted to the subject by Carrel and Crile, and to the much improved technique that has resulted from their labours. In the *American Journal of Surgery* Dr. J. A. Hartwell makes a contribution on the subject, and describes a new method of technique which he has developed and successfully carried out several times on the dog and once on the human subject. The method dispenses with a cannula altogether as used by Crile, and at the same time is much simpler than the end to end anastomosis of Carrel. Briefly, the details are as follows: The radial artery of the donor is dissected out for a couple of inches under local anæsthesia, the small branches isolated and tied, and a ligature thrown round the distal end. The wound is covered with a moist warm pad. A vein in the forearm of the recipient is treated similarly, ligatured at the distal end, and divided just central to the ligature. The adventitia is stripped from the cut end, and three sutures of fine silk, impregnated with vaseline passed through the media and intima. The artery is then tied, clamped, and divided just above the ligature. The adventitia is removed from the cut end and a roll of it is slid up along the artery and a silk suture passed through this. The artery is then immersed in liquid vaseline, and by means of forceps passed into the vein, held open by means of the sutures. The two vessels are held together by clamping the silk sutures attached to the two vessels, and the excess of venous circumference is taken up by a lightly applied clamp. The obstructing clamps are then removed, and the flow of blood proceeds.

THE advantages claimed for this method are that there are no dangers of clotting or of air emboli. The rapidity of flow can be controlled by digital pressure on the artery. In discussing further this subject of blood transfusion the author points out the difficulty that has yet to be overcome of estimating the amount of blood transfused. Various means have been suggested, such as measuring the hæmoglobin content and the variations in the blood count, or noting the changes in blood-pressure and pulse rate in the participants; but there are too many unknown factors to give even approximate knowledge in these ways. The simplest method would appear to be weighing the participants before and during the operation; but this would involve a specially devised scale on which the operating-table could be fitted. A further important consideration which enters into the subject of blood transfusion, and which is discussed in another article in the same journal by Drs. Martin Rehling and Richard Weil, is the compatibility of the two bloods of the participants. In a number of instances transfusion has been succeeded by alarming symptoms, and even by the death of the patient owing to the one blood being hæmolytic towards the other. These authors therefore suggest that preliminary tests for hæmolysis should be carried out before transfusion, and they record a case in which a preliminary test showed slight hæmolytic reaction, and the transfusion which followed was accompanied by transient symptoms of hæmolysis (hæmoglobinuria) in the patient.

IN the operation of Appendicectomy ligature of the stump and cauterisation of the exposed mucosa with carbolic acid is a method which is recognised as safe and satisfactory, but it does not appear to have occurred to any surgeon hitherto to apply the same method to the bowel itself in operations involving closure of an intestinal end. This idea has been conceived and carried out by Dr. Howard Lilienthal, of New York, and in the *American Journal of Surgery* he makes a preliminary report upon the subject, with an account of several cases in which the method has been used and has given satisfactory results. The cases include operations of gastro-jejunostomy and of resection of the gut with lateral anastomosis. The intestinal ends are ligatured firmly with heavy silk, considerable constricting force being used, the free stump is well carbolised, and the ends of the ligature are brought out through the abdominal wound. The ligature comes away at about the end of the second week, sometimes later. Of the six cases reported, two ended fatally. One was a case of intestinal obstruction in the last stages, due to a strangulated hernia, and involving resection of about 30 inches of bowel, and post- mortem examination showed neither leakage nor septic reaction at the stump. The second was a case of gastro-jejunostomy for duodenal ulcer, and post-mortem there was found to be a duodenal leak, with peritonitis and bile in the abdominal cavity. The failure in this case is ascribed by the author to faulty technique. He is of opinion that in the haste necessitated by the patient's condition the wall of the intestine was pierced by the ligature passer.

---

In the *Deutsch. Zeitsch. F. Chir.*, Jungling gives an account of his experiences in the Treatment of Enlarged Prostate by injections of heterogeneous serum derived from pigs, sheep, and cows. The author has followed out the suggeston of Bier, who advocates a trial of the treatment on the grounds that the subsequent histolysis or aseptic dry gangrene ought to favour the resorption of the hypertrophied tissues, and consequently diminish the volume of the organ. This histolytic property of certain of the blood-ferments has already been utilised in the treatment of malignant tumours. The defibrinated blood is injected by means of a long needle directly into the prostatic and peri-prostatic tissues, the needle being guided by a finger placed in the rectum. The needle is inserted in three or four different places, some 2½ c.c. of the serum being injected into each place, not more than 10 c.c. being injected altogether. The strictest asepsis must of course be maintained throughout these manipulations. The operation is repeated three or four times at intervals of eight to ten days. The injections are followed by some pain, tumefaction, and slight hyperæmia. The author has treated fifteen cases in this way. Out of these, seven derived little, if any, benefit, four who had acute retention were improved (one being cured altogether), one died of uræmia, and the remaining three were able to pass urine naturally.

---

Payr, of Greifswald, has published in the *Archives für Klin. Chirurg.* the results of three operations which are somewhat of a surgical curiosity, the technique of which is founded on Carrel's work on the Grafting of Blood-vessels. The idea of draining the ventricles of the brain in hydrocephalics has already led to the devising of several operations to that end, but such success as has been obtained has been transitory. Payr's operation consists in removing one of the internal saphena veins from the leg of the patient and transplanting it in such a manner that there is a direct communication between one of the ventricles and the longitudinal sinus. To avoid reflux of the blood into the ventricle, the resected vein is implanted obliquely into the sinus and the lumen of vein narrowed somewhat. The author has performed this operation on three occasions. The first case, that of a child of 14 months, died two hours after the operation. The second patient, aged seven months, died 14 days after operation, the autopsy revealing a thrombosis in the implanted vein with a partial necrosis of its ventricular end. The third case, that of a child nine years of age, lived somewhat longer. The operation was performed in December 1907, and the second ventricle was drained by a second operation two months later. The child improved for the three months following, but then started vomiting, fell into coma, and died. At the autopsy the longitudinal sinus was found normal, and the two implanted veins permeable.

---

Marcel Ferrand, of Paris, gives a full account of his researches into the nature of Infantile Erythemata. According to this author, in most cases these erythemata clinically and anatomically closely resemble the lesions of eczema. In certain regions secondary causes, such as friction, urinary and fæcal irritation, and secondary infections may modify their appearance; but it is always possible to discover the fundamental morphological and histological characters of the eczema group. Between the simple erosive and the papular syphilide varieties, Ferrand describes transitional forms which all show the same anatomical characters, the difference lying merely in the degrees of intensity of the local reaction. In none of the so-called papular syphilides was any lesion discovered characteristic of syphilis, and search for the treponema pallidum proved negative. Moreover, clinical examination may point to the same result. If there is no induration, if the rash is present on prominent parts and absent in the folds of the body, if it has appeared suddenly and rapidly after a change of diet or a gastro-intestinal disturbance, if the evolution is rapid and the rash disappears completely and quickly with ordinary care as to cleanliness, and if there are no other signs of hereditary syphilis present, the rash is not syphilitic. Recent work of Ravaut has shown that there is frequently a lymphocytosis present in fluid drawn from the spinal meninges of a syphilitic. Ferrand, however, is of opinion that it is not safe to rely on lumbar puncture as a means of diagnosing between a syphilitic papular eruption and a simple one, as a meningeal lymphocytosis is often present in cases of infantile dermatitis apart from syphilis.

---

Hereditary Atrophy of the Papilla, an affection which owes its name to Raymond and Kœnig, is of interest from the medico-legal standpoint, and by reason of its special features is worthy to rank as a morbid entity. The disease is characterised by a partial blindness, which rarely proceeds to complete amaurosis, and is the result of a lesion to the maculo-papillary bundle of the retina. It is always the result of atavistic heredity, either direct or collateral. It is homochronous—that is to say, it starts at about the same period of life in all cases, and it is similar in the sense that in the same family it is always reproduced in the same form. Consanguinity, syphilis, and the various zymotic infections would seem to have no ætiological importance as regards the disease. Tuberculosis, which has been present in some cases, is not sufficient to explain the ordinary lesions and symptoms. The prognosis in this disease is less unfavourable than in the other forms of atrophy of the optic nerve. Patients retain a variable degree of excentric vision, as well as the perception of light.

